# Crystal Structure of EHEC Intimin: Insights into the Complementarity between EPEC and EHEC

**DOI:** 10.1371/journal.pone.0015285

**Published:** 2010-12-16

**Authors:** Yong Yi, Ying Ma, Feng Gao, Xuhu Mao, Hao Peng, Youjun Feng, Zheng Fan, Guihua Wang, Gang Guo, Jinghua Yan, Hao Zeng, Quanming Zou, George F. Gao

**Affiliations:** 1 Department of Clinical Microbiology and Clinical Immunology, College of Medical Laboratory, Third Military Medical University, Chongqing, China; 2 CAS Key Laboratory of Pathogenic Microbiology and Immunology, Institute of Microbiology, Chinese Academy of Sciences, Beijing, China; 3 The 306th Hospital of PLA, Beijing, China; 4 Graduate University, Chinese Academy of Sciences, Beijing, China; 5 China-Japan Joint Laboratory of Molecular Immunology and Molecular Microbiology, Institute of Microbiology, Chinese Academy of Sciences, Beijing, China; 6 National Laboratory of Biomacromolecules, Institute of Biophysics, Chinese Academy of Sciences, Beijing, China; 7 Beijing Institutes of Life Science, Chinese Academy of Sciences, Beijing, China; Charité-University Medicine Berlin, Germany

## Abstract

Enterohaemorrhagic *E. coli* (EHEC) O157:H7 is a primary food-borne bacterial pathogen capable of causing life-threatening human infections which poses a serious challenge to public health worldwide. Intimin, the bacterial outer-membrane protein, plays a key role in the initiating process of EHEC infection. This activity is dependent upon translocation of the intimin receptor (Tir), the intimin binding partner of the bacteria-encoded host cell surface protein. Intimin has attracted considerable attention due to its potential function as an antibacterial drug target. Here, we report the crystal structure of the Tir-binding domain of intimin (Int188) from *E. coli* O157:H7 at 2.8 Å resolution, together with a mutant (IntN916Y) at 2.6 Å. We also built the structural model of EHEC intimin-Tir complex and analyzed the key binding residues. It suggested that the binding pattern of intimin and Tir between EHEC and Enteropathogenic *E. coli* (EPEC) adopt a similar mode and they can complement with each other. Detailed structural comparison indicates that there are four major points of structural variations between EHEC and EPEC intimins: one in Domain I (Ig-like domain), the other three located in Domain II (C-type lectin-like domain). These variations result in different binding affinities. These findings provide structural insight into the binding pattern of intimin to Tir and the molecular mechanism of EHEC O157: H7.

## Introduction

*Escherichia coli* (*E. coli*), is a facultative anaerobe which was originally isolated from the human gastrointestinal tract[1]. Based upon the potential for virulence, this kind of Gram-negative bacteria, can be divided into two major groups: pathogenic *E. coli* and avirulent *E. coli* [1], [2]. Pathogenic *E. coli* has been recognized as the zoonotic agents responsible for a wide spectrum of infectious diseases (e.g., diarrhea, sepsis, and meningitis)[2], [3], [4], [5]. Currently, it is well accepted that the pathogenic *E. coli* can be classified into 5 members that consist of enterohemorrhagic *E. coli* (EHEC), enteropathogenic *E. coli* (EPEC), enteroaggregative *E. coli* (EAggEC), enteroinvasive *E. coli* (EIEC) and enterotoxigenic *E. coli* (ETEC)[1], [6]. Among them, EHEC may be the leading causative agent for sporadic cases and even epidemics of severe *E. coli* infections, posing a great concern to public health worldwide [7]. To our knowledge, two large scale EHEC outbreaks have been recorded (one in Japan, 1996[8] and the other in China, 1999–2000[9]). Moreover, more than 70,000 human cases of EHEC infection with characteristics of diarrhea occur in the United States each year[10].

Among the pathogenic *E. coli* strains, EHEC O157:H7 has been recognized as one of the most notorious pathogens featuring the properties of an extremely common and virulent serotype, and is responsible for a series of severe gastrointestinal illnesses with life-threatening consequences in North America, Europe, China, and Japan[7], [8], [9], [10]. Considering its high pathogenicity, EHEC O157:H7 has been listed as a potential bio-weapon in many countries[11]. In order to understand and control the severe infection of EHEC O157:H7, many research groups have carried out comprehensive investigations at multiple levels ranging from the epidemiology, molecular bacteriology, to the protein interactions between the bacterium and its host[12].

Like most mucosal pathogens, infection of EHEC O157:H7 follows a common cycle: colonization at the mucosal sites, evasion of the host defense, proliferation and host damage[13]. Obviously, there are many virulence factors (e.g., Shiga toxin[14] and intimin[15]) or pathogenicity islands (PAIs) identified to be involved in the general virulence of EHEC[16]. It is worth noting that both the intimin and translocated intimin receptor (*Tir*) genes located on a PAI of ∼43 kb in length (also called locus of enterocyte effacement (LEE)) have been demonstrated to be responsible for the generation of the A/E lesion[15].

Intimin, an out membrane protein expressed by EHEC and EPEC, is required for intimate attachment to the host cell and formation of the A/E lesions [17]. Tir is a bacterial protein which injects into the host cell through the Type III secretion system (T3SS) to function as a receptor specific to intimin[17], [18]. The binding of intimin to Tir mediates the adhesion between the pathogen and its host cell[19]. Shortly after the successful binding, the translocated Tir protein triggers additional signal transduction and actin polarization in host cells, which are essential for lesion formation[20], [21], [22].

Currently, intimin is classified into a large family of adhesin proteins that are capable of evoking A/E lesions and are generally divided into five types (α, β, γ, δ and ε) on the basis of their divergent C-terminus domains[23], [24]. The intimin of EHEC O157:H7 is of γ-type (designated intimin-γ), whereas that of EPEC is intimin-α. In addition, Tir protein of EHEC O157:H7 has also been found to be different from that of EPEC, especially in its pattern of phosphorylation pattern after infiltration of host cells [17]. This implies that the function and structures of EHEC and EPEC intimins may vary to some extent[17]. As we know to date, crystal structure of EPEC Intimin-Tir complex, NMR and crystal structures of the EPEC intimin binding domain alone have been determined [25], [26]. These data gives insight into the molecular mechanism of EPEC adhesion[26]. Moreover, relevant critical amino acids for binding have also been elucidated[12], [26].

Here, we determined the crystal structures of Tir-binding domain of EHEC O157:H7 intimin at 2.8 Å, together with an Asn to Tyr mutant at amino acid 916 (IntN916Y) at 2.6 Å. Complex model of EHEC Intimin-Tir is built, and four key residues involved in their binding are analyzed. Moreover, the differences between the Tir-binding domain of EHEC intimin and that of EPEC are further investigated. This suggests that the EHEC and EPEC intimins can cross complement each other with different binding affinities.

## Results

### Overall Structure of EHEC Int188

Crystals of EHEC Int188 and IntN916Y diffract X-rays at 2.8 Å and 2.6 Å, respectively. Both crystal structures were solved by molecule replacement with the template of EPEC Int188 (PDB code: 1F00). R-free values were separately refined to 29.6% and 26.8%. These two versions of intimin (Int188 and IntN916Y) were crystallized in space group C2 and P2_1_2_1_2_1_, respectively. For the native version, Int188 consists of four molecules in the asymmetric unit while the IntN916Y is present in two molecules per asymmetric unit.

**Figure 1:**
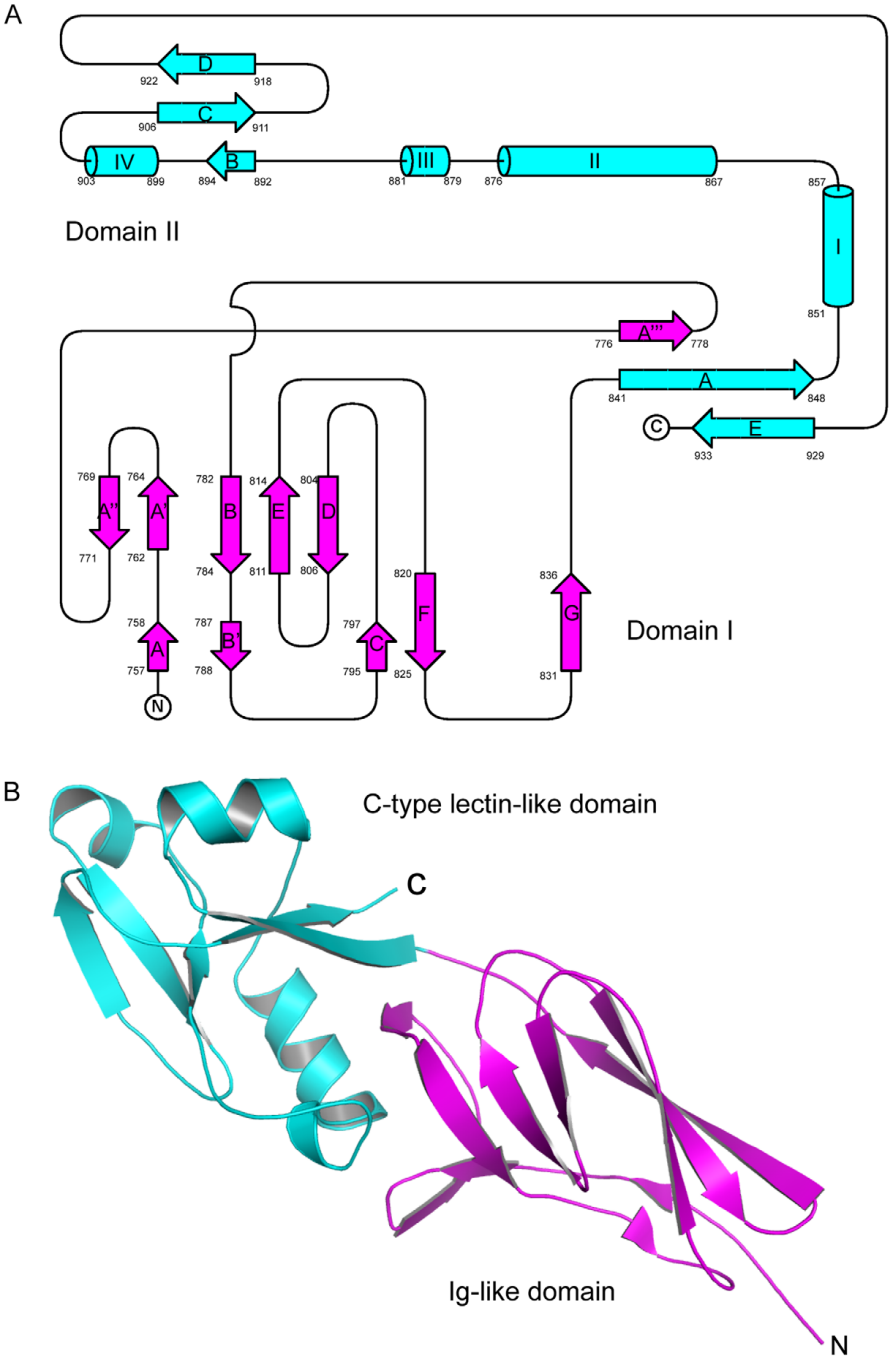
Topological and structural characterization of EHEC Int188. **A)** A topological diagram[33] of EHEC Int188. EHEC Int188 consists of two domain, domain I contains eleven anti-parallel beta-sheets (A, A′, A″, A″′, B, B′ C, D, E, F and G) and ten coils, colored in magentas. Domain II is comprised of two anti-parallel beta-sheets (A, B, C, D and E) and four alpha-helices (I, II, III and IV), marked in cyans. B) An overview of the crystal structure of the EHEC Int188. The structure of EHEC Int188 consists of two domains, domain I (Ig-like domain) is shown in magenta while domain II (C-type lectin-like domain) is shown in cyans.

Topological analysis suggest that Int188 is composed of sixteen beta-sheets together with four alpha-helices (Figure 1A), which can be supported by the crystal structure (Figure 1B). Both topological and structural evidence indicate that Int188 can be obviously categorized into two independent domains (Domain I& Domain II) (Figure 1A, 1B). Further structural blast revealed that these two domains correspond to an Ig-like domain and a C-type lectin-like domain (Figure 1B), respectively. The Ig-like domain at the N-terminus of Int188 is composed of beta-sheet sandwiches which contain eleven anti-parallel beta-sheets (A, A′, A″, A″′, B, B′, C, D, E, F and G) and ten coils. Beta-sheets A′ and A″ between strands A and A′″ extend a platform on top of Domain I that contacts Domain II, helping to define the relative orientation of the two domains. A C-type lectin-like domain was found to be located at its C-terminus, comprised of two anti-parallel beta-sheets (B, C, D and A, E) spaced by four alpha-helices (I, II, III and IV). C932 in the C-terminal strand E forms a disulfide bond with the C858 in helix I. Strand E and strand A in N-terminus of Domain II form the principal strands of the first sheet. The second sheet comprising strands B, C and D, is oriented roughly perpendicular to the first one, such that strands B and E are proximal (Figure 1B). Int188 and IntN916Y behave completely the same at the level of higher structure (not shown).

### The binding pattern of intimin and Tir are similar between EHEC and EPEC

**Figure 2:**
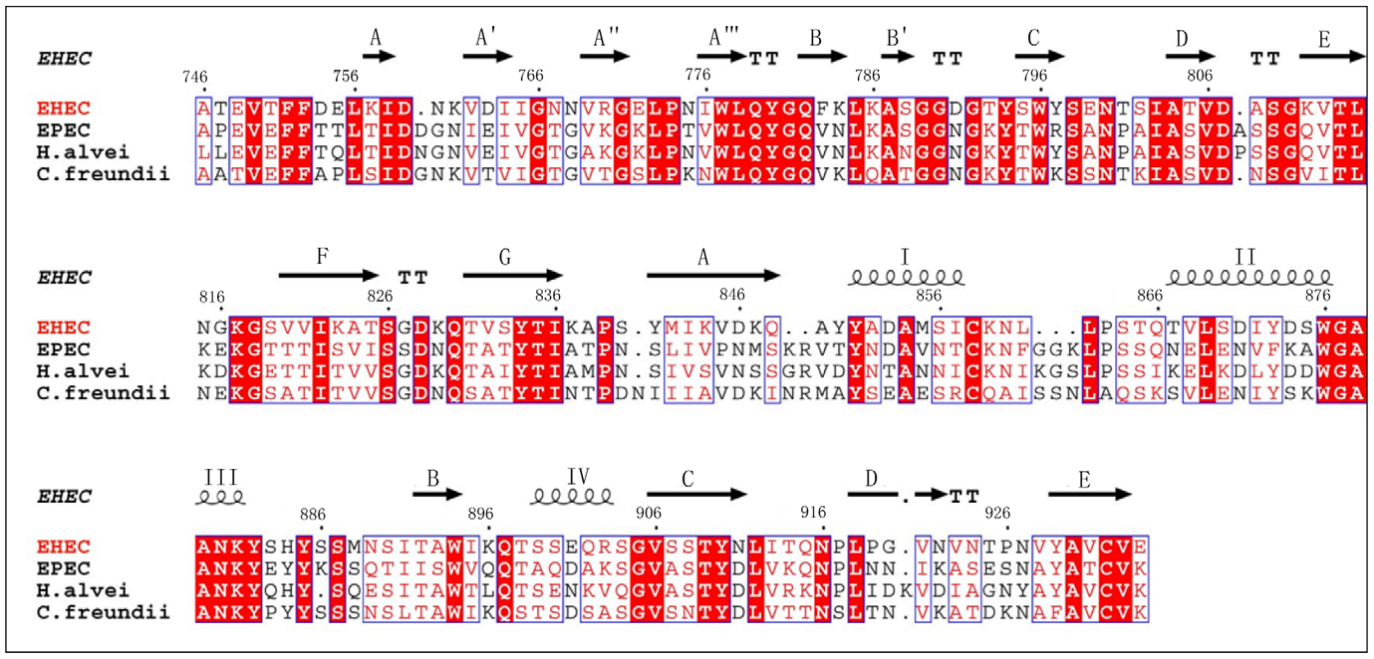
Amino acid sequence alignments of EHEC Int188 with related proteins. Amino acid multiple alignments of EHEC Int188 with its related proteins from different pathogens (EPEC, *Hafnia alvei*, and *Citrobacter freundii*). The residues conserved in all proteins are highlighted in red. Secondary structure elements of EHEC Int188 are shown above the sequence.

**Figure 3:**
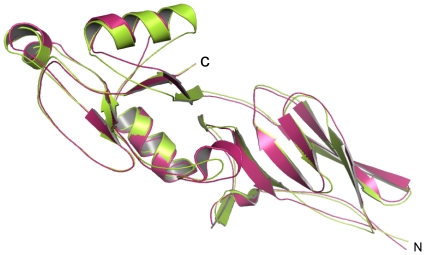
Superposition of Int188 between EHEC and EPEC. Superposition of EHEC Int188 and EPEC Int188 is shown in cartoon. EHEC Int188 is indicated in warm pink, while EPEC Int188 is marked in limon.

Multiple alignments show that EHEC Int188 share the highest sequence identity with three other proteins (EPEC, *H. alvei*, and *C. freundii*) (Figure 2). BLAST analysis reveals that although EHEC Int188 is of 48% sequence identity to that of EPEC, the secondary structures of both proteins are similar in the topological characterization (Figure 2). Furthermore, the superposition demonstrated that Int188 tertiary structures of EHEC and EPEC are highly similar, with an RMSD of 1.2 Å calculated with program Dali [27] (PDB 1F00) (Figure 3). The Tir of EHEC and EPEC share 71% sequence identity, and the critical residues required for binding to intimin are relatively conserved.

**Figure 4:**
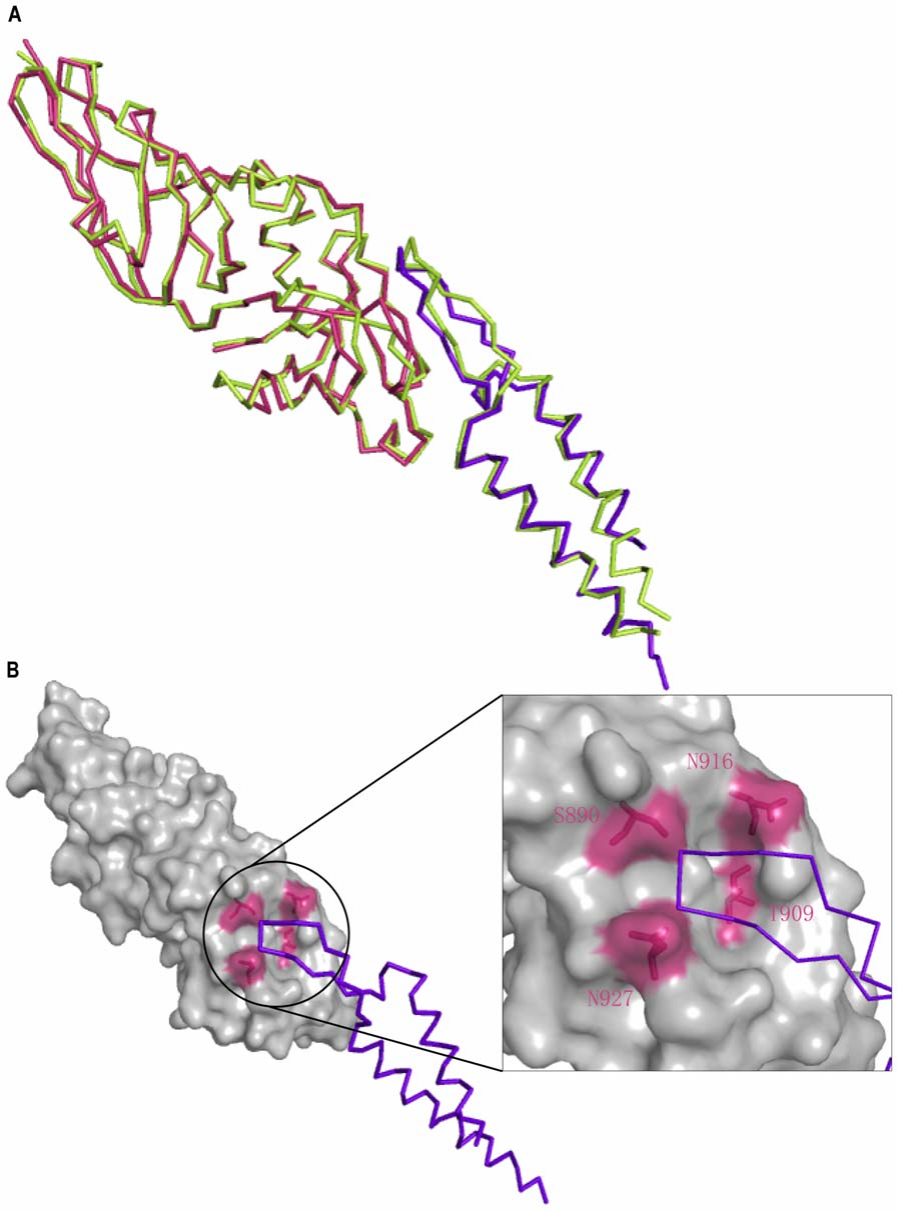
Structural modeling of EHEC intimin-Tir complex and superposition of intimin-Tir complex between EHEC and EPEC. A) Superposition of intimin-Tir complex between EHEC and EPEC. The crystal structure of EPEC intimin-Tir complex is shown in limon. The EHEC intimin and Tir are indicated in warm pink and purple-blue, respectively. B) Structural modeling of EHEC intimin-Tir complex. Intimin is shown as surface in gray. Four important residues (S890, T909, N916 and N927) are marked in warm pink. Tir is shown in purple-blue ribbon. The region involved in the intimin-Tir binding is enclosed in a black circle, in which the details of the key residues are marked in warm pink.

Previous study indicated that EHEC and EPEC intimin can cross-complement each other *in vitro* [17]. According to the structure of EHEC intimin we solved here and the structure of EPEC Intimin-Tir complex (1F02), we built the structural modeling of an EHEC intimin-Tir complex, showing a similar binding pattern between EHEC and EPEC (Figure 4A).

Recent study have demonstrated that four important residues (S890, T909, N916 and N927) of EHEC intimin are essential for Tir recognition[12]. Our structural modeling of the EHEC intimin-Tir complex confirms this claim. It clearly indicates that these four residues locate in the Tir binding pocket which is formed by B, C, D β-sheets and the loop between D, E β-sheets (Figure 4B).

### Structural Comparisons of intimins between EHEC and EPEC

**Figure 5:**
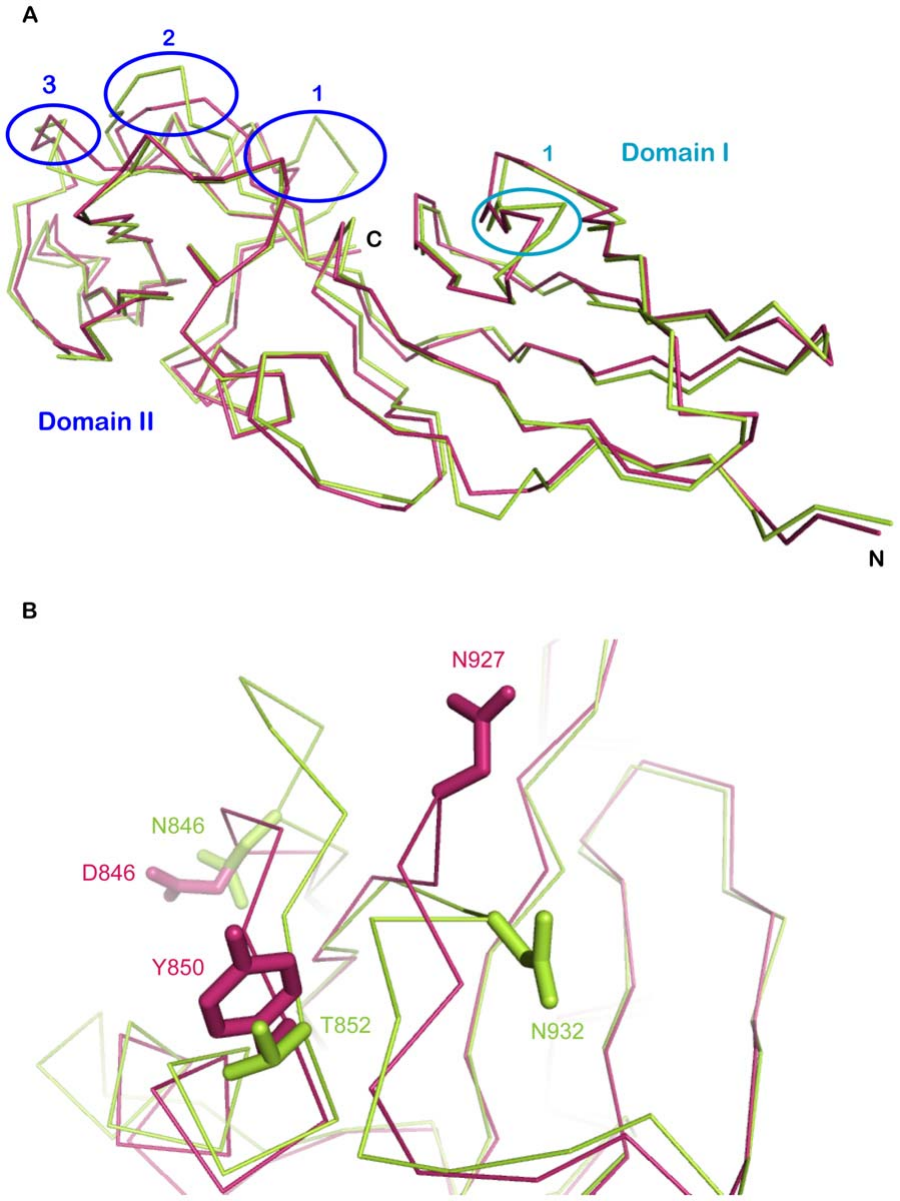
Differences of Int188 between EHEC and EPEC. A) Superposition of EHEC Int188 and EPEC Int188 is shown in ribbon. EHEC Int188 is indicated in warm pink, while EPEC Int188 is marked in limon. Four different variations are highlighted with circles. One of them located in domain I is in teal, the other three located in domain II are indicated in blue. B) Detailed structures of the two differences (2,3) of Int188 between EHEC and EPEC in domain II. EHEC Int188 is indicated in warm pink, while EPEC Int188 is marked in limon.

**Figure 6:**
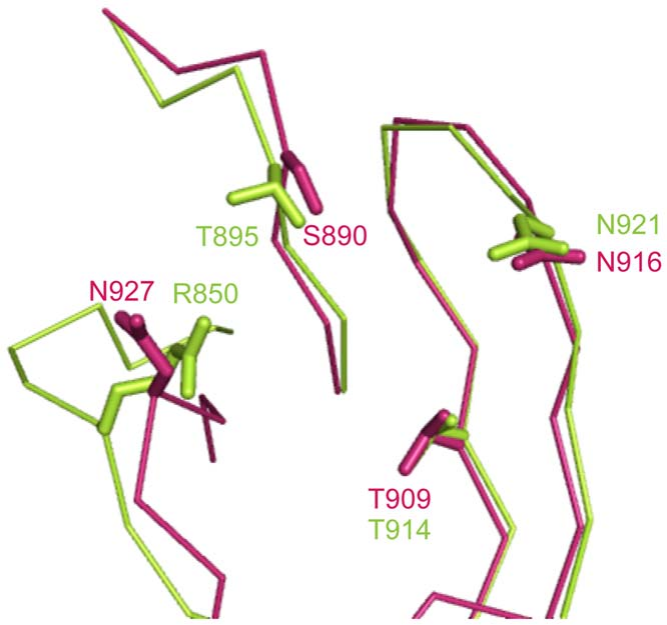
Superposition of four key residues in intimins between EHEC and EPEC. The main chain of EHEC and EPEC intimins are shown as ribbon in warm pink and limon, respectively. S890, T909, N916 and N927 in EHEC intimin are indicated as stick in warm pink, while T895, T914, N921 and R850 in EPEC intimin are marked as stick in limon.

We observed four points of variation based on comparing the structures of intimins between EHEC and EPEC (Figure 5A). For Domain I (Ig-like domain), the main chain of EHEC is quite similar to that of EPEC excluding the region between beta-sheet D and E (Figure 5A). In EHEC, this region forms a regular beta-turn structure consisting of amino acid residues “DASG”, while in EPEC there is an extra S residue at this position, which in turn forms an abnormal loop with amino acid residues “DASSG” (Figure 5A). In Domain II (C-type lectin-like domain), there are three obvious differences between the intimin of EHEC and EPEC. First, the loop between alpha-helix I and alpha-helix II in EHEC spans the region from 859K to 861L, but in EPEC there are three more residues (G, G and K) at this position. Thus, this loop in EPEC is longer than that in EHEC, which contains an extra-loop adjacent to Tir-binding sites (Figure 5A). The second difference in the confirmation of EHEC and EPEC is also found to lie between the A beta-sheet and alpha-helix I. EPEC (residues 846–852) has two more residues than that of EHEC (residues 846–850), in which conformational change directly affect the neighboring main chain (residues 927–933) (Figure 5A). This results in the difference of Tir binding site between EHEC and EPEC. The third major difference in domain II between EHEC and EPEC intimin is at the Tir binding site (Figure 5A, B). In the side chain of EPEC, N932 (equivalent to N927 of EHEC) points to the Tir binding pocket. In contrast, the EHEC N927 in the same position is pointed away from the binding pocket. Additionally, superposition reveals that the distance between the alpha-C atom of this N932 residue in EPEC and the alpha-C atom of the N927 residue in EHEC is 4.75 Å (Figure 5B). Furthermore, structural analysis shows that T909 and N916 in EHEC intimin are identical to T914 and N921 in EPEC, while S890, N927 in EHEC intimin is equivalent to T895 and R850 of EPEC intimin on steric conformation (Figure 6). This suggests that EHEC and EPEC intimin are interchangeable with each other but with different affinities when they bind to Tir[17].

## Discussion

In this study, we solved the structure of EHEC Int188 and its mutant IntN916Y. And we built the structural modeling of an EHEC intimin-Tir complex according to the structure of intimin-Tir complex in EPEC. These data suggested that tertiary structures between intimins of EHEC and EPEC are highly similar, though the sequence identity is relatively low at the amino acid level. The structural modeling indicates that EHEC intimin in complex with its receptor, Tir, produced a similar binding pattern to EPEC and four critical amino acid residues (S890, T909, N916 and N927) of EHEC intimin are considered to be essential for Tir recognition. Specifically, two of them (T909, N916) are identical to the corresponding residues in EPEC, while the other two residues (S890, N927) are equivalent to residues (T895, R850) in EPEC on steric conformation. This suggests that EHEC and EPEC intimin are interchangeable with each other when they bind to their Tir.

We compared with the structure of intimins between EHEC and EPEC. Four points of variation were observed. One is in Domain I (Ig-like domain), the other three are found in Domain II. These variations indicate that EHEC and EPEC intimins cross complement each other with different binding affinities.

For the mutant, IntN916Y, the folding mode is completely the same as the native version, which indicates that this amino acid substitution fails to influence the alpha-C backbone in Int188.

In summary, this report shows the crystal structure of EHEC intimin. The availability of structural information suggests a comprehensive understanding of the interchangeable intimin between EHEC and EPEC, and provides insight into the structure-based design of small molecule drugs utilized to combat against EHEC and EPEC infections.

## Materials and Methods

### Cloning, expression and purification

The Int188 DNA was amplified with bacterial genomic DNA which was isolated from the EDL933 of EHEC O157:H7 strain as a template by PCR. The primers (int-F: 5′- GAA TTC *CATATG* GCG ACT GAG GTC ACT-3′, int-R: 5′-CCG *CTCGAG* TTA TTC TAC ACA AAC-3′) were designed to amplify extra-domains of *eae* (intimin-γ). The amplified *eae* gene was digested with *Nde* I and *Xho* I and cloned into pET-21a vector (Novagen). A mutant, pET-21a-IntN916Y, was also inadvertently obtained in this experiment. The recombinant plasmids were verified by DNA sequencing.

### Protein Expression and Purification

Both of these two recombinant proteins (Int188 and IntN916Y) were expressed as inclusion bodies and they were then lysed using a sonicator and centrifuged at 16,000 g. The pellet was washed three times with a solution of 20 mM Tris-HCl, 100 mM NaCl, 1 mM EDTA, 1 mM DTT and 0.5% Triton-100. Refolding of the purified inclusion bodies was carried out as described earlier[28] with minor modifications[29] The refolded protein was then purified using Resource Q anion exchange chromatography followed by Superdex 75 size exclusion chromatography.

### Crystallization and Structure Determination

Crystals of Int188 and IntN916Y were obtained by the hanging-drop vapor diffusion method at 291K. Initial screening was performed using crystal screen I and II (Hampton Research). A 1 µl droplet of protein solution (5, 10, and 15 mg/ml, respectively) mixed with equal amount of reservoir solution was equilibrated against 200 µl of reservoir solution. Crystals were first observed in 4 days with the No. 22 condition of Crystal Screen II (12% PEG20,000, 0.1 M MES, pH 6.5). After several rounds of optimization, one of the more promising crystallization conditions was optimized (15% PEG20,000, 0.1 M MES, pH 6.5) at 277K.

Crystal diffraction data were collected in house on a Rigaku MicroMax007 rotating-anode X-ray generator operated at 40 kV and 20 mA (Cu Kα; λ = 1.5418 Å) equipped with an R-AXIS VII++ image-plate detector. The crystals were flash-frozen in liquid nitrogen after addition of 15% (v/v) glycerol to the mother liquor, mounted in nylon loops and flash-cooled in a cold nitrogen-gas stream at 100 K using an Oxford Cryosystem with reservoir solution as the cryoprotectant. Crystals of EHEC Int188 and IntN916Y diffracted at 2.8 Å and 2.6 Å, respectively. Data were processed and scaled with Crystalclear[30].

The crystal structures of Int188 and IntN916Y belong to the space group C2 and P2_1_2_1_2_1_, respectively. The coordinates of EPEC intimin (PDB 1F00) [26] were used to serve as an initial model for IntN916Y using the program CNS[31]. The refinement was performed using simulated annealing, energy minimization, restrained individual B factor and the addition of water molecules in the CNS program. The respective working R_working_ and R_free_ dropped to 21.3% and 26.8% for all data from 50 to 2.6 Å. Subsequently, the structure of Int188 was solved by molecular replacement using the IntN916Y molecule as a search model. After the same refinement steps, the working R_working_ and R_free_ dropped to 25.3% and 29.6% for all data from 50 to 2.8 Å. The final structures of Int188 and IntN916Y were checked for geometrical correctness with PROCHECK[32] and analyzed and compared with EPEC intimin-α (PDB 1F00) using programs from the CCP4 package[33] as well as the molecular graphics programs Coot[34]. The refinement statistics of structure are given in Table 1. The atomic coordinates and the structure factors for both Int188 and IntN916Y have been deposited in the Protein Data Bank, where they have been assigned the identifiers 3NCW and 3NCX.

**Table 1:** X-ray diffraction data collection and refinement statistics of intimin crystals.

	Molecular type	
Parameters	Int188	IntN916Y
A. Data collectionSpace group	C2	P212121
Resolution limit(Å)	2.80 (2.90−2.80)^a^	2.60 (2.69−2.60)^a^
Cell dimensions	*a* = 235.16 *b* = 44.81 Å, *c* = 129.12 Åα = γ = 90°, β = 97.53°	*a* = 43.74 Å *b* = 92.37 Å *c* = 100.01 Åα = β = γ = 90°
Total reflection	109738	82591
Unique reflection	32396	12232
Completeness(%)I/σR_merge_ (%)B. Refinement	96.3(92.7)4.1(2.4)23(43.2)	99.7(100)9.6(4.5)13.2(35.6)
*R*-factor	25.3%	21.3%
*R*_free_ b	29.6%	26.8%
r.m.s. deviations		
Bond lengths (Å)	0.008	0.003
Bond angles (°)	1.65	0.618

a Values in parentheses are given for the highest resolution shell.

b R_free_ is calculated over reflections in a test set (5%) not included in atomic refinement.
